# A Scoping Review of Available Tools in Measurement of the Effectiveness of Conservative Treatment in Lipoedema

**DOI:** 10.3390/ijerph19127124

**Published:** 2022-06-10

**Authors:** Monika Czerwińska, Jacek Teodorczyk, Rita Hansdorfer-Korzon

**Affiliations:** 1 Department of Physiotherapy, Medical University of Gdańsk, 7 Dębinki Street, 80-211 Gdańsk, Poland; rita.hansdorfer-korzon@gumed.edu.pl; 2 Department of Nuclear Medicine and Radiology Informatics, Medical University of Gdańsk, 17 Mariana Smoluchowskiego Street, 80-214 Gdańsk, Poland; jacek.teodorczyk@gumed.edu.pl

**Keywords:** lipoedema, lipoedema, complete decongestive therapy, women’s health, physiotherapy, conservative treatment

## Abstract

(1) Background: Due to insufficient knowledge of lipoedema, the treatment of this disease is undoubtedly challenging. However, more and more researchers attempt to incorporate the most effective lipoedema treatment methods. When assessing a new therapeutic method, choosing correct, objective tools to measure the therapeutic outcome is very important. This article aims to present possible instruments that may be used in the evaluation of therapeutic effects in patients with lipoedema. (2) Methods: The data on therapeutic outcome measurements in lipoedema were selected in February 2022, using the Medical University of Gdansk Main Library multi-search engine. (3) Results: In total, 10 papers on this topic have been identified according to inclusion criteria. The tools evaluating the therapeutic outcomes used in the selected studies were: volume and circumference measurement, body mass index, waist-to-hip ratio, ultrasonography and various scales measuring the quality of life, the level of experiencing pain, the severity of symptoms, functional lower extremity scales, and a 6 min walk test. (4) Conclusion: The tools currently used in evaluating the effectiveness of conservative treatment in women with lipoedema are: volume and circumference measurement, waist-to-hip ratio, body fat percentage, ultrasonography, VAS scale, quality of life scales (SF-36, RAND-36), symptom severity questionnaire (QuASiL), Lower Extremity Functional Scale and 6 min walk. Choosing a proper tool to measure the treatment outcome is essential to objectively rate the effectiveness of therapeutic method.

## 1. Introduction

Lipoedema is a chronic disease of subcutaneous adipose tissue resulting in the pathological proliferation of adipose tissue, mainly around the lower extremities [1]. It mostly affects women, and the beginning is usually associated with puberty, pregnancy, or menopause [2]. Lipoedema is still not entirely understood [3]. Currently, the combination of genetic predisposition and hormonal changes is thought to be responsible for the occurrence of lipoedema, but the exact etiology is not yet established [2]. It is estimated that lipoedema prevalence among the general public is 1:72,000, but these data have also not been fully confirmed [4].

In recent studies, Michelini et al. discovered that mutation in the AKR1C1 gene can be related to the development of lipoedema [5]. Hormonal disturbances are also thought to be connected to lipoedema. Potentially, there is a disbalance between oestrogen receptors (ER alpha and ER beta) in adipose tissue of lipoedema affected certain areas, occurring as a result of faulty expression of oestrogen receptors. It can explain the abnormal proliferation of adipose tissue in the affected areas [6,7].

A study by R. Crescenzi et al. has shown that there is an increase in the sodium content in the skin among lipoedema patients, which is a hallmark of inflammation thought to be responsible for pain in lipoedema [8]. 

The distribution of adipose tissue in lipoedema is always bilateral and symmetric, and feet remain unaffected [1,2,9,10]. Moreover, women with lipoedema experience painful ailments such as sensitivity on palpation, easy bruising and heaviness in the legs [6,11,12].

Insufficient knowledge of lipoedema and lack of specific diagnostic criteria often lead to incorrect diagnosis as obesity or lymphoedema [13]. It is usually a reason for delayed diagnosis and absence of the correct treatment which is associated with further disease progression and a worsening of the patient’s condition [14].

At the moment, lipoedema is considered an incurable disease, and the proposed therapeutic options are mainly aimed at reducing symptoms and preventing their progression [15]. The current treatment of lipoedema is based on therapy methods normally used in the treatment of lymphedema [16]. Conservative treatment includes complex decongestive therapy (CDT) constituted of manual lymphatic drainage (MLD), compression therapy, skincare and physical exercises, and intermitted pneumatic compression (IPC) [17,18]. Other methods such as subcutaneous adipose tissue manual therapy and ketogenic diet are currently under examination [19,20,21,22]. Liposuction is the surgical treatment of lipoedema [23].

MLD is mainly aimed at draining the intracellular fluid from swollen areas. In lipoedema, there is no significant accumulation of intracellular fluid; however, it is believed that MLD, due to its anti-oxidative effect, could help reduce pressure sensitivity and pain in patients suffering from lipoedema [6]. Compression therapy is aimed at improving venous and lymphatic flow and at supporting the tissues and improving the shape of the limbs. In addition, compression improves mobility, facilitates movement, increases functionality and, due to its anti-inflammatory effect, can reduce pain [24]. Intermitted pneumatic compression (IPC) is a method of mechanical massage employing special air-inflated cuffs to increase the lymphatic flow [25].

Another important therapeutic element is a broadly understood movement therapy. Particularly recommended are low-intensity aerobic exercises, exercises in water and breathing exercises. The therapeutic process should focus on the patient’s limitations, needs and expectations, while the main focus is reducing the patient’s subjective ailments [3,16].

Due to the lack of distinct guidelines for lipoedema treatment, more and more new methods are being tested for their effectiveness. Evaluation of the efficiency of therapy should be objectively measured and be reproducible, therefore, specific measurement methods are crucial in examining various therapeutic methods in lipoedema patients treatment [17,18,19,20,21,25,26,27,28,29].

This study aims to review and evaluate the use of various measurement tools in assessing the effectiveness of conservative lipoedema treatment. The secondary aim is to present the currently available therapeutic methods in lipoedema treatment.

## 2. Materials and Methods

**Figure 1:**
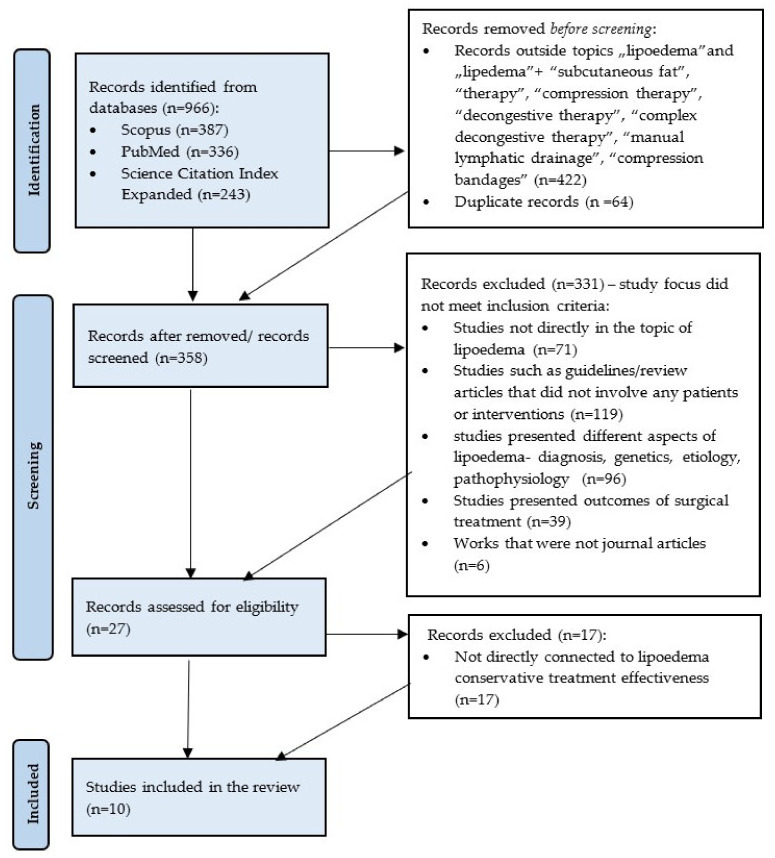
Flow diagram adapted from PRISMA presenting the process of identifying and screening of the articles for inclusion and exclusion.

A review of currently available literature was carried out on 8 February 2022, using the Medical University of Gdansk main library multi-search engine. The initial research consisted in searching for journal articles in English focused on the terms “lipoedema” or “lipedema” available in the following databases: Scopus, Medline Complete (PubMed) and Science Citation Index Expanded. After an initial search, 966 results were obtained, (Scopus: 387; PubMed: 336; Science Citation Index Expanded: 243) and the results were narrowed to the terms “lipoedema”, “lipedema”, “subcutaneous fat”, “therapy”, “compression therapy”, “decongestive therapy”, “complex decongestive therapy”, “manual lymphatic drainage” and “compression bandages” and 544 results were collected. After removing duplicates, 358 journal articles were included in the title and abstract screening. The authors decided to remove a total number of 330 works: 71 not connected to the topic of lipoedema; 96 addressing different aspects of the lipoedema such as genetics, etiology, diagnosis, pathophysiology; 119 publications that did not involve any patients or interventions such as guidelines or review articles; 39 presenting outcomes of surgical treatment; and 6 works that were not journal articles. Then, 27 articles were assessed for suitability. A total number of 10 publications were selected on the basis of the specified inclusion criteria: (1) Journal articles available in the full-text format; (2) Publications presenting clinical trials or case studies; (3) Studies involving patients with lipoedema; (4) Research assessing conservative treatment methods; (5) Studies evaluating the effectiveness of treatment by objective tools. The PICOS components (Population, Intervention, Comparison, Outcome, Study Design) were used to generate a research question: What are the objective and effective tools to evaluate the therapeutic outcome (O) of conservative treatment (I) by comparing the pre- and post-intervention results (C) in lipoedema patients (P). Studies included in this study were clinical trials and case studies. The research was based on PRISMA methodology (Figure 1). The review was conducted according to the PRISMA method. The papers included in the review are presented in Table 1. 

## 3. Results

### 3.1. Volume Reduction

Seven studies reported volume measurement as a tool to assess the effectiveness of conservative lipoedema treatment methods [17,20,21,25,26,27,28]. Since in the majority of cases the lipoedema applies to the lower part of the body, only the lower extremity measurements were pursued. Various techniques were used to obtain the volume. The most frequently used method was the Kuhnke disc method (presented in five publications) [17,20,21,25,28]. In his technique, the volume of the lower limb is obtained by the collection of the circumference of the extremity in 4 cm intervals starting from the ankle and finishing at the highest point of the inner thigh [17,28]. Two of the studies, which used the abovementioned method, aimed to evaluate the effectiveness of subcutaneous adipose tissue manual therapy in lipoedema patients [20,21]. Subcutaneous adipose tissue (SAT) manual therapy is a deep tissue massage concentrated on fat, muscles and fascia to improve tissue quality [20,21]. The results showed significant volume reduction after a 4-week therapeutic program—on average, 2 L of reduction in the right leg and 1 L in the left in research conducted by Herbst, and 0.9 L volume reduction in a study by Ibarra [20,21]. (Table 2) A study of 38 women with lipoedema conducted by Szolnoky in 2008 showed a significant volume reduction (0.9 L) in women who participated in a 5-day complex decongestive physiotherapy (CDP) program in comparison to the control group not receiving any specific therapy (0.1 L of reduction) [28]. In another study by Szolnoky, the application of complex decongestive physiotherapy with or without intermittent pneumatic compression (IPC) was assessed. Volume reduction was observed in both groups, but it was slightly higher in a group with added IPC (1.4 L in left leg, 1.1 L in right leg), in comparison to the CDP group (1.1 L of reduction in left leg, 0.8 in right leg) [17]. The last research study which used the Kuhnke disc method as a tool to determine leg volume was conducted by Atan et al. [25]. The research aimed to determine whether the complex decongestive therapy (CDT) combined with exercises was more effective than the intermittent pneumatic compression combined with exercises in comparison to the application of exercises only. A decrease in the lower extremity volume could be observed among all participants. The biggest reduction was recognised in the CDT and exercise group (1.2 L in both right and left legs). The difference in volume reduction was not substantially different in the IPC and exercise (0.5 L left and 0.7 L right limb) and exercise-only groups (0.6 L on both sides), but the result was still significant for the general outcome [25] (Table 2).

The research presented by Volkan-Yazıcı Melek et al. obtained the volume changes using Perometer 400NT [26]. The device is thought to provide a fast and reliable technique of limb volume measurement using infrared light. Perometer 400 is composed of a square frame in which the assessed extremity is placed. During the assessment, the diameter measurements were obtained using certain reference points in 4.7 mm intervals. These measurements are used to calculate the volume. The results of the study showed that after 24 days of the therapeutic program with manual lymphatic drainage, compression therapy and intermittent pneumatic compression, the mean volume reduction at 0.4 L and 0.7 L could be observed in the left and the right leg, respectively [26] (Table 2).

In case studies presented by A.C.M. Amato, the volume changes were obtained using bioimpedance. It is a method employing weak electric current to indicate body composition, body fat and muscle mass. The volume reduction in the lower limbs of three patients after 5, 8 and 11 months of MLD, aquatic exercises and an anti-inflammatory diet was significant—1.9 L, 1.2 L and 10.7 L, respectively [27]. 

Table 2 presents the level of volume reduction in lower extremities after conservative treatment among lipoedema patients.

**Table 2:** The effects of complete decongestive therapy (CDT) in lipoedema treatment assessed by volume measurement.

Ref.	Number of Subjects	Subjects Excluded	Duration	Measurement Method	Intervention	Mean Volume Prior Intervention	Mean Volume after Intervention	Reported *p* Value
[26]	23	0	5 sessions per week for on average 24 days	Perometer 400 NT	MLD, Compression therapy, IPC	**Right**-13.5 L **Left**-13 L	**Right**-12.8 L **Left**-12.6	**Right** < 0.001 **Left** < 0.001
[25]	33	2	5 sessions a week for 6 weeks	Kuhnke’s disc method	CDT and exercise (*n* = 11)	**Right**-11.9 L **Left**-12 L	**Right**-10.7 L **Left**-10.8 L	**Right** < 0.001 **Left** < 0.001
					IPC and exercise (*n* = 10)	**Right**-10.9 L **Left**-10.5 L	**Right**-10.2 L **Left**-10 L	**Right**-0.022 **Left**-0.031
					Exercise only (*n* = 10)	**Right**-12.3 L **Left**-11.9 L	**Right**-11.7 L **Left**-11.3 L	**Right**-0.028 **Left**-0.023
[20]	7	0	12 × 90 min sessions of SAT therapy over 4 weeks	Kuhnke’s disc method	SAT manual therapy	**Right**-13 L **Left**-13 L	**Right**-12 L **Left**-11 L	**Right** < 0.007 **Left** < 0.002
[17]	23	0	5 days—one treatment daily	Kuhnke’s disc method	CDT (*n* = 13)	**Right**-17.7 L **Left**-17.9 L	**Right**-16.9 L **Left**-16.8 L	**Right**-0.0011 **Left**-0.001
					CDT and IPC (*n* = 10)	**Right**-15.3 L **Left**-15.4 L	**Right**-14.2 L **Left**-14 L	**Right**-0.000958 **Left**-0.00039
[27]	3	2	8 months	Bioimpedance	MLD, aquatic exercises, anti-inflammatory diet	19.6	18.4	<0.001
			5 months			23.7	21.8	<0.001
			11 months			32.5	21.8	<0.001
[21]	7	0	12 × 90 min sessions of SAT therapy over 4 weeks	Kuhnke’s disc method	SAT manual therapy	12.9	12	0.007
[28]	38	0	5 days—one treatment daily	Kuhnke’s disc method	CDT (*n* = 21)	16.5	15.6	<0.05
					Control group—no treatment (*n* = 17)	15.5	15.4	N/A

### 3.2. Waist-to-Hip Ratio, Body Mass Index and Weight

Weight measurement, body mass index (BMI) and waist-to-hip ratio are commonly used tools to evaluate outcomes of slimming. Studies presenting data on those tools in lipoedema treatment were identified to investigate whether they are beneficial in evaluating the effectiveness of conservative treatment in lipoedema. 

The waist-to-hip ratio is a ratio of the circumference of the waist to the circumference of the hips. It can be used to determine a disproportion between the torso and lower limbs, which is a characteristic feature of lipoedema [20]. The authors identified four studies reporting on WHR changes after conservative treatment in lipoedema patients [19,20,21,25]. All of the presented WHR values were less than 1 which could be expected of a gynoid-type of female body shape. Even though there were some changes in body weight in three studies, it ought to be noted that none of the presented studies showed a significant difference in WHR after the treatment independent from a therapy method employed [19,20,21,25] (Table 3).

One study reported BMI changes after a conservative treatment program. There was a significant difference in BMI in a group with CDT and exercises and a group with exercises only. The group with the application of intermittent pneumatic compression did not show any significant changes in BMI [25] (Table 3).

### 3.3. Circumferences

In his study, Schneider compared the efficacy of manual lymphatic drainage with or without low-frequency vibrotherapy in a group of 30 women with lipoedema [29]. In both cases there was a decrease in circumference; however, the women after MLD with vibrotherapy presented a greater reduction. In the MDL group, the circumferences of the ankles, calves and thighs decreased by 0.2, 0.6 and 1.8 cm, respectively. On the other hand, the patients who attended MLD and vibrotherapy noted a reduction of 1.2, 1.8 and 2.6 cm in the ankles, calves and thighs, respectively. The circumference of the feet did not change significantly post-treatment [29].

Another study used circumference measurement in five reference points including the smallest circumference of the ankle, the largest circumference of the calf, the smallest below the knee, mid-patellar circumference and circumference in the middle of the thigh. The reduction of 4.5% and 1.9% was noted in the left and right thigh, respectively. The mid-patellar circumference decreased by 0.4% on the left and 1.2% on the right extremity and the below-knee measurement showed a reduction of 1.6% on the left side and 0.23% on the right side. The calf circumference decreased by 2.2% on the left and 3.1% on the right leg. The ankle circumference did not change substantially [26].

In the case study of women with lipoedema, the circumferences of the upper extremity, the torso and the lower extremity were obtained pre- and post-treatment. All of the measurements decreased after the 22-month diet program, but the biggest reduction could be observed in the hips (27.98%), the thighs (27.03%), the arms (26.14% left and 24.42% right) and waist (23.85%) [19] 

### 3.4. Bioimpedance and Dual X-ray Absorptiometry

Bioimpedance was used in three studies to measure therapeutic outcomes. Bioimpedance is a method of measuring body composition using electric current. R. Connataro et al. conducted body fat percentage (Bf%) analysis using bioimpedance in one lipoedema patient who had followed a ketogenic diet program. Initially, Bf% was about 47% and at the 22-month follow-up it was 27% [19]. 

Two remaining studies reported the usage of both bioimpedance and dual X-ray absorptiometry scan (DXA scan). DXA is originally used to measure bone density; nonetheless, it can be also used to indicate body composition and fat content [20,21]. A study by Ibarra aimed to use bioimpedance as a tool to evaluate body fluids in lipoedema patients. The results showed a significant decrease in total body water from 35.6 kg at baseline to 34.9 kg post-treatment. The same study used DXA to indicate total body mass and fat mass. Following the subcutaneous adipose tissue therapy, the leg fat mass decreased significantly from 17.8 to 17.4 kg. Other analysed features did not change significantly [21]. 

In comparison, Herbst et al. used bioimpedance to measure body weight, fat mass and muscle mass. The muscle mass increase could be observed post-treatment (47.6 kg at baseline, 50.7 at the end of the study) and the fat mass decreased from 19.7 to 17.8 kg. Mean body weight did not significantly change (90 at baseline, 90.7 at the end of the study). In the same study, DXA was used to measure the total body mass, the torso fat and the leg fat. None of the above features changed significantly post-treatment [20].

### 3.5. Ultrasonography

Ultrasonography was used to measure the effects of therapy in two studies [20,21]. Both studies were aiming to assess the effectiveness of SAT therapy in people with lipoedema. In studies conducted by Ibarra et al., ultrasonography was performed on the lower abdomen and lower extremities (anterior calf, lateral thigh, calf, posterior thigh and inner thigh) to assess the tissue consistency. In total, six out of seven patients presented at least one (min = 1, max = 8) hyperechoic mass in the calf or thigh region. The total number of hyperechoic mass instances in the patients before treatment was 22. After the SAT therapy, all issues of the previously found masses had been resolved, but seven (min = 1, max = 2) new masses in a total of five patients were identified at the end of the study. The mass accounted for was, on average, 0.7 cm under the skin, and the dimensions were 1.3 by 0.8 cm. Moreover, four of the assessed women had visual fluid accumulation prior to treatment which has been reduced after SAT therapy [21]. 

In a study by Herbst, ultrasonography was performed on the lower abdomen and the thighs. The ultrasound evaluation revealed that the tissue was more fibrotic in three patients at baseline in comparison to the status at the end of the study [20].

### 3.6. Visual Analogue Scale

The Visual Analogue Scale (VAS) is a commonly used tool in measuring the level of experienced pain. It is a 10-point numerical scale, in which a patient is asked to indicate their level of pain by choosing a number (0—no pain at all; 10—maximum pain). Four studies provided information on the pain level before and after the treatment [18,20,21,25]. Significant pain decrease could be observed in two cases [18,25]. Szolnoky et al. presented a pain reduction of 2.55 points after conservative therapy [18]. Accordingly, Atan et al. reported a mean reduction of 3.4 points on the VAS scale regardless of the conservative treatment method [25] (Table 4).

The two remaining studies did not report any significant change in the level of pain after SAT therapy [20,21]. The results of the pain level measurements are presented in the Table 4. 

### 3.7. Quality of Life

The level of quality of life and health was measured in three studies [25,28,29]. Schneider conducted a study involving 30 female lipoedema patients. The quality-of-life level was defined to evaluate the effectiveness of manual lymphatic drainage with or without low-frequency vibrotherapy. The questionnaire used in the assessment was “Quality of life with Chronic Disease”, and it was focused on the following dimensions: physical performance, ability to relax, positive mood and negative mood. Each item included possible answers based on a 5-point-Likert scale—very bad, bad, moderate, good and very good. After both MLD and MLD with vibrotherapy treatment, an improvement in quality of life could be observed; however, patients undergoing manual lymphatic drainage with vibrotherapy experienced a greater (23%) improvement in quality of life, compared to an 8% improvement in patients undergoing MLD only [29].

In Cannataro’s study, presenting the case of women with lipoedema undergoing a ketogenic diet plan, a RAND-36 questionnaire was used to investigate the quality of life. All of the aspects of the questionnaire improved significantly after the 22-month treatment: Physical Functioning from 60/100 to 85/100; Role Limitations Due to Physical Health from 25/100 to 100/100; Role Limitations Due to Emotional Problems from 35/100 to 100/100; Energy/Fatigue from 30 to 55/100; Emotional Well-Being from 35 to 65/100; Social Functioning from 75 to 100/100; Body Pain from 30 to 75/100; and General Health from 20 to 60/100 [19]. 

Atan et al. assessed the patients’ health status employing the Short Form 36 Health Survey (SF-36), which is composed of dimensions similar to the abovementioned RAND-36. A total number of 31 patients were assigned to three different therapeutic groups: group 1 underwent complete decongestive therapy with an exercise program, group 2 had intermitted pneumatic compression therapy and an exercise program and group 3 participated only in the exercise program. Considerable improvement in all of the groups could be observed in the following aspects: Physical Functioning (69% group 1, 36% group 2, 41% group 3), Social Functioning (51.5% group 1, 60% group 2, 34% group 3), Health Change (92% group 1, 62% group 2, 43% group 3) and Pain (89% group 1, 127% group 2, 57% group 3). The increase in Emotional Well-Being could be observed in group 1 and group 3 with the improvement of 46% and 24%, respectively. General Health, Role Limitations Due to Physical Health and Energy/Fatigue were enhanced only in the group 1 with 89%, 180% and 66% improvement, respectively. The Role Limitations Due to Emotional Problems did not change post-treatment in any of the groups [25]. 

### 3.8. Experiencing Symptoms

The severity of lipoedema symptoms was assessed in four case reports described by Amato, with the use of the Lipedema Symptom Assessment Questionnaire (QuASiL) created by Rapprich and adapted by Amato [30,31]. The questionnaire consists of 15 specific questions about lipoedema-related ailments, and the patients are asked to rate the severity of their symptoms on a 0–10 scale, where 0 means no ailment and 10 is maximum severity. In all of the cases, conservative treatment in the form of manual lymphatic drainage, aquatic exercises and an anti-inflammatory diet was introduced. The first patient scored 89/150 in QuASiL at baseline and after an 8-month therapeutic program the score reduced to 58/150. In the second case report, the therapy only lasted a 1 month, but the QuASiL decreased from 44 to 11/150. The next patient scored 103 on the QuASiL score at the beginning, and after 5 months of treatment, the score dropped to 22/150. The last patient scored 115 points at baseline, and after 11 months of therapy the score declined to 59/150. It should be noted that despite the length of the treatment, the Lipedema Symptom Assessment Questionnaire showed a significant reduction in ailments in all the cases [27].

### 3.9. Functional Scales

The Lower Extremity Functional Scale (LEFS) was used in two studies [20,21]. The LEFS scale consists of 20 questions aiming at the evaluation of the lower limbs function. In every question, the patient is rated from 0—extreme difficulty to 4—no difficulty. Herbst et al. reported a 17% improvement in LEFS after 4 weeks SAT therapy program [20]. However, in a more recent study, Ibarra et al. noted a 14.3% decrease in LEFS score after 4 weeks SAT therapy program [21].

Atan et al. in their study of 31 women with lipoedema used a 6 min walk test to evaluate the functional capacity and therapeutic response. During the test, the patient is asked to walk as long a distance as possible in 6 min. The therapeutic techniques in the study included MLD, IPC and exercises. Independent of the used method of treatment, the 6 min test improved from 318.22 m at baseline to 371 m at the end of the study [25]. 

## 4. Discussion

Since the knowledge of lipoedema pathophysiology is not complete, the treatment remains a challenge [3,25,32]. Moreover, an additional difficulty is the late diagnosis and delayed introduction of the therapy. Two separate studies by Romejin et al. and by Bauer et al. revealed that the span between the first lipoedema symptoms and a diagnosis is on average 18.3 years and 15 years, respectively [14,33]. It generally causes the development of an advanced stage of lipoedema, and as a result, serious functional impairments, which cannot be reversed. Since the diagnosis is made based on physical examination and patient’s history, knowledge of lipoedema characteristic features is crucial to distinguish it from other diseases such as lymphoedema and obesity [9]. Lymphoedema is more often asymmetrical, does not cause bruising and pain and it is caused by fluid accumulation. Accordingly obesity does not cause pain and bruising and the fat tissue is distributed evenly throughout the body, contrary to lipoedema [34].

Furthermore, a large proportion of lipoedema patients is thought to also to be obese [35]. Dudek et al. in their study of 329 lipoedema patients, only 4.3% of subjects had normal body weight while obesity was present in 81.3% of patients [36]. In a different study of 129 lipoedema patients by Romejin, obesity was identified in 61.9% of participants [33]. Obesity in women with lipoedema can aggravate the symptoms of the disease and decrease the patient’s mobility [6]. Thus, striving for a healthy body weight in lipoedema patients is crucial, and reducing body mass would definitely benefit the patient’s condition [6].

The results of our analysis show that regardless of the treatment method, the number of study participants or the duration of the intervention in all the presented cases, the lower extremity volume was reduced after conservative treatment [17,20,21,25,26,27,28]. It can suggest that limb volume measurement could be a beneficial tool in evaluating the effectiveness of lipoedema treatment. The reliability and accuracy of volume measurement using a perometer versus tape measurement were assessed by Sharkey. The results show a high level of accuracy of both methods, and that limb volume measurement is appropriate method in a clinical setting [37]. Although volume measurement is generally beneficial in measuring therapy outcomes, it does not give the researcher the possibility of accurately distinguishing the type of reduced tissue (lipoedema fat, intracellular fluid, muscle mass). It only gives rough information about the general effect of therapy.

Body circumference measurement can demonstrate if the post-treatment reduction was concentrated only on the areas unaffected by lipoedema or if the level of lipoedema tissue was also decreased [38]. Our research showed that, despite the method of treatment, circumferences in lower parts of the body decreased in all included studies. Unfortunately, as in volume measurement, it is also impossible to distinguish the type of reduced tissue. 

Waist-to-hip ratio, indicating body shape, could possibly be perceived as a valuable tool for the therapeutic effect measurement because it shows if the disproportion is growing or diminishing post-treatment. Our study, however, revealed no change in WHR after therapy [20,21].

Ultrasonography is another useful tool indicating therapeutic effects. Studies presented in our research used ultrasound examination to observe the changes in adipose tissue structure after the therapeutic intervention. The results showed a reduction in the number of hyperechotic masses in fatty tissue and a decreased level of fibrosis after treatment. Ultrasonography has been previously described as a tool to support lipoedema diagnosis [39]. The study on 63 women with lipoedema and 27 controls revealed that the thickness of adipose tissue in women with lipoedema was much higher than in healthy women. The thickness of adipose tissue in lipoedema patients was 20.61 mm, 13.31 mm, 16.34 mm and 11.9 mm on the thigh, lateral leg area, pre-tibial region and supramalleoar, respectively, while the controls presented the tissue thickness of 12.5 mm, 6.6 mm, 8.4 mm and 6.35 mm on the same regions [39]. Currently, there are no studies that present tissue thickness measurement to indicate the outcome of therapy, but this method could potentially be an important tool to rate the effectiveness of treatment.

Various types of scales and questionnaires are beneficial in indicating the improvement of patients’ health status [40]. Decreasing the level of pain and severity of symptoms, increasing the quality of life and increasing functionality are very important therapeutic goals, and the evaluation of therapy methods by means of these tools could indicate the most successful treatment for patients [31,35,38]. Five outcome instruments: the Short Form 36 (SF-36), the lymphedema-specific Freiburg Quality of Life Assessment for Lymphatic Disorders (FLQA-lk), the Knee Outcome Survey Activities of Daily Living Scale (KOS-ADL), the Symptom Checklist-90-revised (SCL-90R) and the Six-Minute Walk Test were evaluated in 96 lipoedema and 107 lymphoedema patients in a study by Angst et al. The results showed that all the tools used have favourable features in the specific assessment of lipoedema and lymphoedema, and can be successfully used in the assessment of therapeutic outcomes [40]. 

The main limitation for this study is a low number of clinical trials connected to lipoedema treatment methods. Despite the fact that lipoedema is getting more and more interest among researchers, there are still only a few articles assessing non-operative treatment effectiveness among women with lipoedema. Moreover, most of the studies were not randomised controlled trials, but a different type of trial or case study. Another limitation might be differences between included studies such as different duration time or a diverse number of subjects. 

## 5. Conclusions

Finding a proper tool to evaluate treatment outcome in lipoedema patients is necessary to determine the most effective therapy. 

Circumference and volume measurements are beneficial instruments in general comparisons of therapeutic outcomes; however, they do not provide specific information on the type of reduced tissue. 

Body mass index, body weight and body fat percentage provide broad information on body shape and the patient’s health, and they can be used for an overall assessment of the effects of therapy, but they are focused on the obesity rather than specific lipoedema symptoms. 

Ultrasonography, although not widely used in lipoedema, could be helpful instrument in objectively assessing therapeutic outcomes specific for lipoedema patients, due to the possibility of assessing the tissue consistency. Using ultrasound to compare the thickness of subcutaneous adipose tissue in the specific points pre- and post-treatment should also be considered in future research assessing treatment effectiveness. 

Quality of life and symptom severity questionnaires, the pain severity scale and functional scales are valuable tools for evaluating the patient’s health and can be successfully used in diagnosing lipoedema patients to indicate changes in their health status after therapeutic interventions.

## Figures and Tables

**Table 1:** Description of articles included according to PRISMA.

Article Type	Focus	Reference
Randomised Clinical Trial	Comparing the outcomes of manual lymphatic drainage with or without low frequency vibrotherapy on circumference and quality of life.	[29]
	Comparing effects of complete decongestive therapy or intermitted compression or exercises.	[25]
Clinical Trial	Effects of complete decongestive therapy on volume and circumference.	[26]
	Impact of complete decongestive therapy on limb volume and capillary fragility.	[28]
	The effects of subcutaneous adipose tissue therapy.	[21]
	The influence of lymphoedema treatment on pain and volume in lipoedema patients.	[18]
Pilot Study	Comparing the effectiveness of complete decongestive therapy with or without pneumatic compression.	[17]
	The effect of manual subcutaneous adipose tissue therapy.	[20]
Case Study	Outcomes of conservative treatment in 5 patients with lipoedema.	[27]
	Long-term benefits of ketogenic diet in lipoedema patient.	[19]

**Table 3:** The effects of CDT on therapeutic outcome in lipoedema measured with BMI and WHR.

Ref.	Number of Subject	Duration	Intervention	Weight before	Weight after	Weight Reduction	BMI before	BMI after	BMI *p* Value	WHR before	WHR after	WHR *p* Value
[25]	31	5 sessions a week for 6 weeks	CDT and exercise (*n* = 11)	99.08 kg	97.4 kg	1.68 kg	43.49	42.78	<0.001	0.86	0.85	0.064
			IPC and exercise (*n* = 10)	97.52 kg	96.03 kg	1.49 kg	40.66	39.66	NS	0.82	0.83	0.073
			Exercise only (*n* = 10)	102.27 kg	100.28 kg	1.99 kg	42.06	41.17	0.001	0.84	0.85	0.0792
[20]	7	12 × 90 min sessions of SAT therapy over 4 weeks	SAT manual therapy	90	90.7	+0.7 kg	N/A	N/A	N/A	0.7	0.7	NS
[19]	1	22 months	Ketogenic diet	N/A	N/A	41 kg	N/A	N/A	N/A	0.8	0.86	N/A
[21]	7	12 × 90 min sessions of SAT therapy over 4 weeks	SAT manual therapy	87.6	87.1	1.5	N/A	N/A	N/A	0.78	0.78	NS

**Table 4:** The effects of therapeutic outcome in lipoedema treatment measured with VAS.

Ref.	Number of Subject	Duration	Intervention	VAS Before	VAS after	VAS *p* Value
[25]	31	5 sessions a week for 6 weeks	CDT and exercise (*n* = 11)	7.73	3.09	<0.001
			IPC and exercise (*n* = 10)	8.3	4.9	0.001
			Exercise only (*n* = 10)	7.9	5.7	0.002
[20]	7	12 × 90 min sessions of SAT therapy over 4 weeks	SAT manual therapy	1.3	0.7	0.005
[18]	38	5 days—one treatment daily	Manual lymphatic drainage, intermitted pneumatic compression	5.89	3.34	0.001
[21]	7	12 × 90 min sessions of SAT therapy over 4 weeks	SAT manual therapy	2.1	1.3	NS

## Data Availability

The data presented in this study are openly available in PubMed, Scopus and Science Citation Index Expanded.
